# Case Report: PFAPA (Periodic Fever, Aphthous Stomatitis, Pharyngitis, and Cervical Adenitis) Syndrome With a Novel TNFAIP3 Mutation

**DOI:** 10.1002/iid3.70178

**Published:** 2025-03-12

**Authors:** Jiewen Deng, Hui Guo, Ruina Kong, Jie Gao

**Affiliations:** ^1^ Department of Cardiovascular Diseases The First Affiliated Hospital of Naval Medical University Shanghai China; ^2^ Department of Pharmacy, Shanghai Tenth People's Hospital Tongji University School of Medicine Shanghai China; ^3^ Department of Rheumatology and Immunology The First Affiliated Hospital of Naval Medical University Shanghai China

**Keywords:** adenitis, adult‐onset of PFAPA, aphthous stomatitis, case report, periodic fever, pharyngitis, TNF alpha induced protein 3

## Abstract

**Background:**

Periodic fever, aphthous stomatitis, pharyngitis, and cervical adenitis (PFAPA) syndrome has been considered as a childhood syndrome. Its etiopathogeny is unknown however, currently considered as auto‐immune inflammatory disease. Recently, a few cases of adult‐onset of PFAPA syndrome have been reported. However, there is no report about the adult‐onset of PFAPA case with a novel *TNFAIP3* Mutation.

**Objective and Method:**

Followed by detailed clinical inquiry, related laboratory tests, genetic sequencing and treatment, we reported a case with the adult‐onset of PFAPA syndrome with a novel TNFAIP3 mutation.

**Results:**

We have found a novel mutation in the gene *TNFAIP3* in an adult patient with periodic fever, aphthous stomatitis, pharyngitis, and adenitis—the PFAPA syndrome, under the environmental factor‐COVID‐19 vaccination.

**Conclusion:**

This case demonstrated adult‐onset of PFAPA symptoms, including periodic fever of unknown origin, which can occur in adult patients with the familial hereditary *TNFAIP3* mutation and environmental factors. And the therapeutic measures provide some reference and practical significance for the treatment of PFAPA syndrome.

## Summary

This case demonstrated adult‐onset of PFAPA symptoms, including periodic fever of unknown origin. There is first report about the adult‐onset of PFAPA case with the novel TNFAIP3 Mutation. The patients developed adult‐onset of PFAPA symptoms under the environmental factor‐COVID‐19 vaccination. The therapeutic measures provide some reference and practical significance for the treatment of PFAPA syndrome

**Abbreviations**
ACMG: American College of Medical Genetics
ANCA: anti‐neutrophil cytoplasmic antibodies
Anti‐IL‐6: anti‐interleukin‐6
anti‐β2‐GP1: anti‐β2‐glycoprotein 1
BD: Behcet disease
COVID‐19: corona virus disease 2019
CRP: C‐reactive protein
CT: computed tomography
DNA: nucleic acid (DNA) detection
EB virus IgM: Epstein‐Barr virus immunoglobin M
FDG: fluorodeoxyglucose
GM: galactomannan
HIV: human immunodeficiency virus
IFN‐stimulated: interferon‐stimulated
IFN‐α: interferon‐α
IgG 4: immunoglobulin G4 subtype
IL‐1: interleukin‐1
IL‐10: interleukin‐10
IL‐12P70: interleukin‐12P70
IL‐17A: interleukin‐17A
JAK: Janus kinase
NSAIDs: nonsteroidal anti‐inflammatory drugs
PET‐CT: positron emission tomography‐computed tomography
PFAPA: periodic fever, aphthous stomatitis, pharyngitis, adenitis
SLE: systemic lupus erythematosus
STAT‐1: signal transducer and activator of transcription‐1
TB: tuberculosis
*TNFAIP3*
: TNF alpha induced protein 3

## 1 Introduction

Periodic fever, aphthous stomatitis, pharyngitis, and adenitis (PFAPA) syndrome [1, 2, 3] is a self‐limiting autoinflammatory disease characterized by recurrent episodes of fever lasting 3–7 days, which occur every 2–8 weeks. These episodes are often accompanied by symptoms such as stomatitis, pharyngitis, and cervical adenitis. The exact cause of PFAPA syndrome is unknown, but it is believed to have a complex genetic basis with environmental influences. The diagnostic criteria for PFAPA syndrome are specific and include several factors. These factors include negative blood cultures, no history of sick contacts in the family, absence of typical upper respiratory tract infection symptoms like cough or runny nose, failure to respond to antibiotics, and a positive response to a single dose of corticosteroids [2, 4].

Here, we presented a case of an adult patient with periodic fever, aphthous stomatitis, pharyngitis, and adenitis and conformed to the diagnostic criteria for PFAPA syndrome under the environmental factor‐corona virus disease 2019 (COVID‐19) vaccination. Interestingly, the patient's genetic testing result uncovered a heritable variant of uncertain significance (c.63A>T (P.I21=) variant in the *TNFAIP3* gene) making no difference in the amino acid composition of proteins. There is no report about the adult‐onset of PFAPA case with the novel TNFAIP3 Mutation. Hence, the special case deserves to be reported and explored.

## 2 Case Presentation

**Figure 1:**
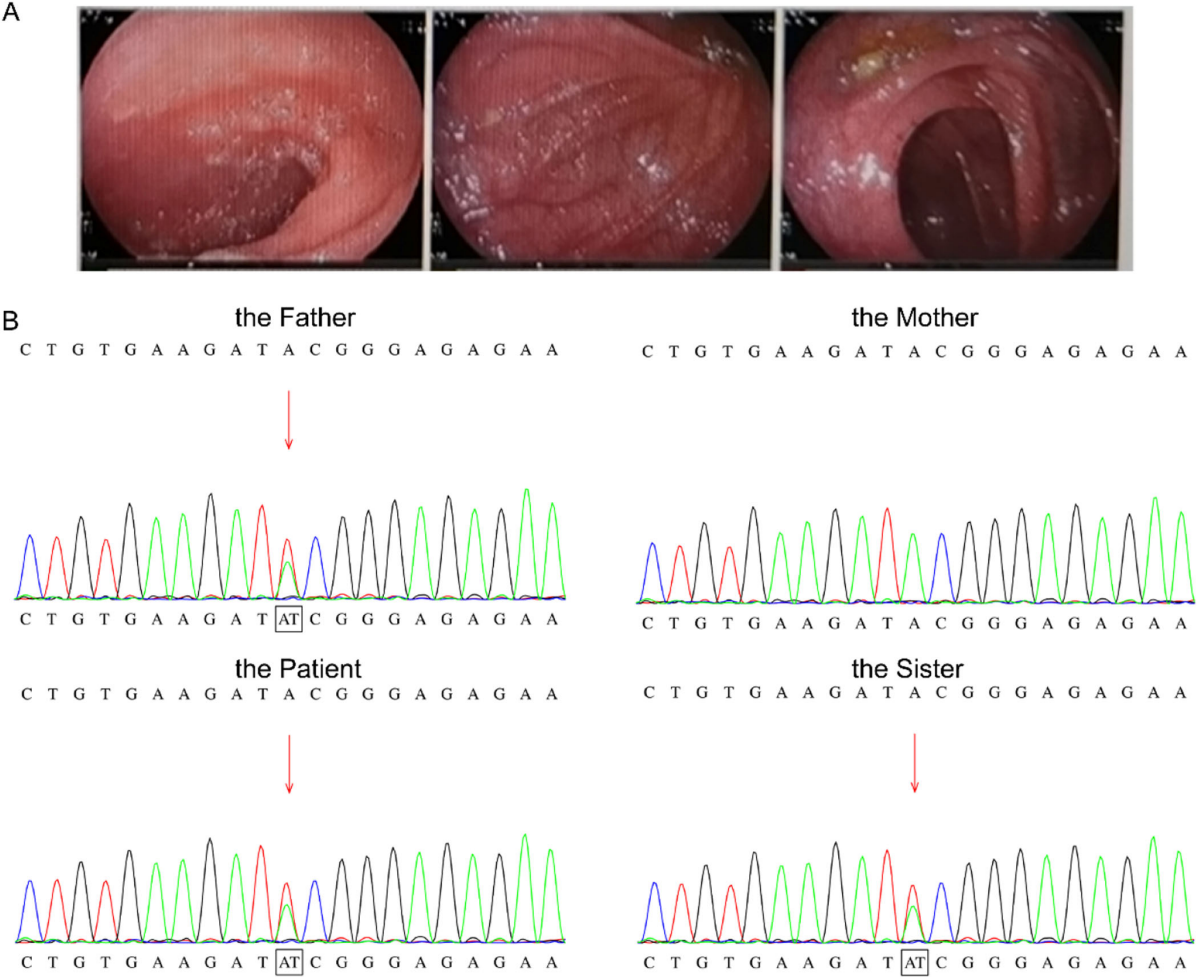
(A) There were no obvious abnormalities under col‐colonoscopy. (B) Heterozygous variant in TNFAIP3 gene c.63A>T(p. I21 = ) was detected by genetic testing from the samples of the patient, his father and his sister. While no variant in the TNFAIP3 gene was found in the sample of the patient's mother.

A 25‐year‐old young male patient had an intermittent fever for more than 1 year without obvious inducement. Each fever lasted for 4–5 days, and the body temperature gradually rose in the afternoon, reaching the peak at about 39°C, mostly accompanied by sore throat, oral ulcer and neck tubercle. The symptoms were periodic, and the cycle was about 40 days. On April 30, 2021, the patient had a fever with a maximum of 39.4°C without obvious inducement, accompanied by pharyngeal discomfort, headache, and no other positive symptoms, and the body temperature returned to normal after 4–5 days of fever. After vaccination (June 4, 2021), the patient developed fever again for 4 days in June 9, 2021, with a maximum body temperature of 39°C, and self‐administered “ibuprofen” retreated fever. The patient had an intermittent fever of up to 40.2°C, cycle after 40 days, more in the middle of each month, that was accompanied by pharyngeal discomfort, oral ulcer, neck, swelling, tenderness, occasional left leg pain, left leg pain, left thumb, small finger pain, and with multiple visiting in the local hospital. The blood routine results suggested that white blood cells and inflammation (CRP, blood) slightly increased, meanwhile, antinuclear antibodies, anti‐neutrophil cytoplasmic antibodies (ANCA), anti‐cardiolipin antibodies, and anti‐β2‐GP1 antibodies were negative during fever. Dosing cephalosporin and amoxicillin for anti‐infection treatment effects were not good, and the temperature returned to normal after 4–5 days of fever. On October 14, 2022, the patient had a fever again with a temperature of 39.7°C, with a sore throat, oral ulcer, and neck lymph node swelling and tenderness. For further treatment, he was admitted to our department with a “fever pending investigation”. On the day of admission, physical cooling and the treatment of loxonin were not effective. The temperature of the patient returned to normal after changing to dexamethasone 5 mg. On the morning of October 15, several red papules appeared on the left thigh, gathering into pieces. Two days later, the patient developed a red rash at the left back of the waist, with a tingling sensation, slightly itchy, and some visible blisters, and the rash was banded, with a sore throat and no fever, diagnosed as Herpes zoster by the dermatologist. Then foscarnet sodium 3 g qd for intradermal injection was used for a continuous 7 days, moreover, oral cobamamide 1 mg tid and topical acyclovir ointment and calamine lotion for outward application were applied. After admission, routine white blood cell was counted 11.76 × 10^9^/L ↑, neutrophile granulocyte was 10.69 × 10^9^/L ↑, lymphocytes 7.3％, monocytes 1.7％↓, neutrophils 90.9％↑, inflammatory index CRP7 0.30 mg/L ↑, while flow cytokines was suggested interferon (IFN)‐α 8.98 pg/mL ↑, interleukin (IL)‐10 9.59 pg/mL ↑, IL‐12P70 19.32 pg/mL↑, IL‐17A 42.69 pg/mL ↑, with the decreased T lymphocytes. Ferritin and autoantibody profile, immunofixation electrophoresis, detection of T cells for TB infected, immunoglobulin G4 subtype (IgG 4), tumor markers, fungal d‐glucan detection, the galactomannan (GM) detection, blood coagulation, EB virus nucleic acid detection were indicated no obvious abnormalities. HIV, syphilis, and hepatitis C virus antibodies were all negative and herpes simplex virus, rubella virus, cytomegalovirus, and EB virus IgM antibodies were all negative. Simultaneously, the col‐colonoscopy result showed no obvious abnormalities (Figure 1A), as well as the chest computed tomography (CT). positron emission tomography‐computed tomography (PET‐CT) didn't show an abnormal increase in FDG metabolism. Bone piercing tips indicated the hyperplastic bone marrow image with the negative bone marrow cultures, as well as the negative blood culture and bone marrow smear. Flow and chromosome examination showed no obvious abnormalities. After excluding the fever caused by common connective tissue disease, infection, tumor, drugs, and other causes, with an effective result of glucocorticoid therapy, considering that it was a periodic fever of unknown cause (PFAPA?). We recommend the patient and his parents take high throughput gene sequencing by DNBSEQ‐T7 platform (MGI, China), the gene sequencing data analyzed by the programs such as SIFT, Mutation Taster, PolyPhen‐2, PROVEAN, Human Splicing Finder and Max EntScan. Variants were selected after combining the clinical information and classified according to the American College of Medical Genetics and Genomics (ACMG) guidelines, confirmed by Sanger sequencing. Meanwhile the patient was dosed for loxonin (60 mg, tid) and colchicine (0.5 mg, bid) and then discharged for follow‐up in November 2022. After discharge, the patient was still febrile for 1 week on November 22, up to 38°C. Colchicine was stopped due to intestinal adverse reactions, and then tofacitinib (5 mg, bid) and prednisone (10 mg qd) were altered.

On December 25, 2022, tofacitinib was discontinued due to COVID‐19 infection, and he was treated with loxonin (60 mg, tid) and prednisone (10 mg qd). After 5 weeks of treatment, the patient no longer developed any other symptoms. The heterozygous variant in *TNFAIP3* gene c.63 A>T(p. I21=) was detected in genetic testing (Figure 1B).

## 3 Discussion

In our case, first, the diagnosis of acute lymphoblastic leukemia was ruled out, because the patient's bone marrow examination showed no significant abnormalities and no abnormal lymphoblasts detected. And PET‐CT results made us exclude the diagnosis of malignant tumors. Second, the absence of the clearly identified infectious pathogen led to the exclusion of infectious diseases. Therefore, the Autoimmune disease was taken into consideration. The patient mainly presented with periodic fever, no skin changes, no recurrent vulva ulcer, no ophthalmitis, and negative acupuncture reaction, without consideration of Behcet disease. In addition, the patient was negative in antinuclear antibody profiles, which can also exclude systemic lupus erythematosus. Then according to the diagnostic criteria for adult‐onset still's disease in 1992 [5]. the patient had a fever of more than 39°C, but the fever did not last for more than a week, occasionally with arthralgia and short duration. The white blood cell was counted with a mild increase, not reaching 15 × 10^9^/L. So adult Still disease was excluded.

Meanwhile, the most represented characteristic of the case was the symptom‐ recurrent (or periodic) fevers. Recurrent (or periodic) fevers are characterized by inflammatory flares separated by intervals of general overall well‐being. Some conditions are caused by a genetic defect and are collectively referred to as hereditary recurrent fever (HRF), which need to be excluded for the patient's definite diagnosis. Familial Mediterranean fever (FMF) is caused by mutations of MEFV [6, 7]; mevalonate kinase deficiency (MKD), by mutations of the mevalonate kinase gene (MVK) [8, 9]; tumor necrosis factor (TNF) receptor‐associated periodic fever syndrome (TRAPS), by mutations of type I TNF receptor (TNFSRF1A) [10]; and cryopyrin‐associated periodic syndromes (CAPS), by mutations of NLRP3 [11, 12]. However, in our case, the patient genetic testing result demonstrated not the above‐mentioned referred mutations but a variant of uncertain significance (c.63A>T (p. I21 = ) variant in the TNFAIP3 gene).

Hence the clinical presentation of this patient conformed to the diagnostic criteria for PFAPA syndrome [13], which is a multifactorial disorder [1, 14, 15, 16]. PFAPA syndrome is a multifactorial disorder, closely linked with disease causing gene mutations (rare) or a combination of genetic susceptibility and epigenetic modifications arising from exposure to the environment (more common) [17, 18]. In the case, by high throughput gene sequencing, the patient has a new and firstly found heterozygous variant of TNFAIP3 gene c.63 A > T (p. I21 = ) in exon2. TNFAIP3 gene was associated with familial Behcet's autoinflammatory syndrome type 1 (OMIM 616744), which was identified as autosomal dominant inheritance. According to the ACMG guidelines, the gene variation interpretation was explained as uncertain significance for clinical significance, also detected in the father's and sister's samples (Figure 1B), with the suggestion of heredity. However, his father and sister appeared without any discomfort yet carrying mutant genes, hint of the involvement with environmental factors. Tracing the potential cause, the patient developed symptoms after receiving the COVID‐19 vaccination—the likely acquired environmental influence. Considering the clinical symptoms and signs caused commonly by the inherent and environmental factors, with an effective glucocorticoid treatment, this case had a certain particularity and deserved to be reported.

Previous therapeutic strategies for autoinflammatory diseases such as PFAPA have primarily relied on findings from retrospective studies and the expertise of physicians, including colchicine, nonsteroidal anti‐inflammatory drugs (NSAIDs), glucocorticoids, IL‐1 blockers, anti‐TNF agents, anti‐IL‐6 agents, and Janus kinase (JAK) inhibitors [14, 19]. As is followed in our case, the patient with a febrile state had a timely response to a single glucocorticoid therapy. Because of the Herpes zoster appearance, NSAIDs loxonin and colchicine were applied. Then due to the intolerance to colchicine with the appearance of diarrhea, according to the medication experience, the JAK inhibitor tofacitinib was taken into consideration and eventually took effect. COVID‐19 infection stopped the JAK inhibitor tofacitinib treatment. At last, under the combination application of nonsteroidal drugs and glucocorticoid treatment, symptoms improved and periodic fever symptoms subsided. Post the approximately 4‐month‐treatment, the patient had stopped medication with symptom free. Our case provided the specific example and practical significance to some extent for the treatment and diagnosis of PFAPA syndrome.

## Author Contributions

Jiewen Deng examined and treated the patient. Jie Gao and Ruina Kong designed and supervised this study. Jiewen Deng and Hui Guo wrote the manuscript.

## Ethics Statement

The study on human participants in this research followed the local legislation and institutional requirements, and therefore ethical review and approval were not required.

## Consent

All patients/participants involved in the study provided written informed consent to participate. Additionally, written informed consent was obtained from individuals for the publication of any potentially identifiable images or data included in this article.

## Conflicts of Interest

The authors declare that the research was conducted in the absence of any commercial or financial relationships that could be construed as a potential conflict of interest.

## Data Availability

The raw data will be made available by the authors, without undue reservation.
